# Helping Children to Participate in Human Papillomavirus–Related Discussions: Mixed Methods Study of Multimedia Messages

**DOI:** 10.2196/28676

**Published:** 2022-04-11

**Authors:** Aurora Occa, Hayley M Stahl, Sarah Julien-Bell

**Affiliations:** 1 Department of Communication University of Kentucky Lexington, KY United States; 2 Department of Communication University of Illinois at Urbana Urbana, IL United States

**Keywords:** animation, game, HPV, child-parent communication, child-physician communication, pilot study, children, health communication, communication technologies, vaccination, health education

## Abstract

**Background:**

Human papillomavirus (HPV) can cause several types of cancers and genital warts. A vaccine is available to prevent HPV infections, and several efforts have been made to increase HPV education and, eventually, vaccination. Although previous studies have focused on the development of messages to educate children about HPV and the existence of the HPV vaccine, limited research is available on how to help children better communicate with their parents and health care professionals about the HPV vaccination. In addition, limited research is available on the target audience of this study (Italian children).

**Objective:**

This manuscript describes a study assessing the feasibility of using an evidence-based animated video and a web-based game to help children (aged 11-12 years) participate in discussions about their health—in particular when such conversations center around the HPV vaccination—and improve several HPV-related outcomes. The study also compares the effects of these 2 educational multimedia materials on children’s knowledge and perceptions of HPV prevention.

**Methods:**

A mixed methods approach consisting of focus group discussions and an experiment with children (N=35) was used to understand children’s experiences with, and perceptions of, the animated video and the game and to measure possible improvements resulting from their interaction with these materials.

**Results:**

Both the animated video and a web-based game increased children’s knowledge and positive perceptions about HPV and HPV vaccination. Any single message was not more effective than the others. The children discussed aspects of the features and characters they liked and those that need improvements.

**Conclusions:**

This study shows that both materials were effective for improving children’s education about the HPV vaccine and for helping them to feel more comfortable and willing to communicate with their parents and health care professionals about their health. Several elements emerged that will allow further improvements in the design and development of the messages used in this study as well as the creation of future campaigns.

## Introduction

### Background

Engaging children and preteens in conversations about their health is important for their well-being and future health behaviors [1,2]. However, children and preteens are often not included in such conversations, even when they express a specific interest in being involved, because both parents and health care professionals consider such conversations particularly challenging [3]. Furthermore, research aimed at understanding and improving children’s engagement in health discussions is limited [4-6]. As a consequence, children’s information needs are often unmet, despite evidence showing that children can actively participate in health discussions and decision-making processes [4], as well as benefit from being included in them [1,2,6]. Some health-related conversations are perceived as more challenging by both parents and their children; for example, conversations about sexual health or cancer [7,8]. A conversation concerning HPV vaccinations is one such because it shares barriers faced by both sexual health– and cancer-related discussions. Human papillomavirus (HPV) can negatively affect the human body by causing several types of cancers and genital warts [9]. Today, HPV is known to be the most frequently diagnosed sexually transmitted disease [10]; yet, vaccination hesitancy remains a problem. To limit the risk of contracting an HPV infection, children should receive the HPV vaccine at age 11-12 years [11,12].

Given children’s need to receive health information and the communication challenges they may face, recently researchers developed and assessed several educational strategies targeting in particular preteens and adolescents in the United States [13-15]. In addition to improving attitudes and knowledge, these educational materials seem to support an increased rate of HPV vaccinations over methods that target parents only [14,16]. These promising results and the barriers that children and preteens face to communicate about their health indicate the importance of developing evidence-based interventions and message strategies to educate children about specific health topics such as HPV and support them in discussing these health topics with their parents and health care professionals [17]. These interventions need to take into consideration the cultural background of the children and the perceived social norms because these can influence the way children and adolescents talk about health with their family and the way they conceptualize health [18,19].

Interventions using communication technologies have shown the potential to improve HPV vaccination behaviors [20]. These interventions can allow for customization of information to individuals’ needs [20] and culture [21]. However, only limited research exists on how communication technology can support children’s learning about health [14,22,23]. Even less research is available on the role that communication technology–based interventions can have in empowering children and preteens to discuss their health or the HPV vaccine [7].

### Communication Technologies for HPV Education and Vaccination

Research and interventions about HPV vaccinations have adopted several communication technologies such as videos, Facebook pages, SMS text messages, and emails [20], as well as mobile apps [24,25]. However, when communicating with children, preteens, or adolescents specifically, researchers have tested mainly videos and SMS text messages focused on HPV [14,20], despite the fact that several other strategies have been shown to be promising for educating children about health [23,26,27]. For example, serious games and educational animations can encourage adolescents’ engagement in health decision-making [26,28-33]. Thompson et al [28] developed and assessed a serious videogame about the HPV vaccine for adolescents. The game increased adolescents’ likelihood of receiving the vaccine and aided the participants in personal health decision-making. Similarly structured videogames have also been used for other health-related behavior changes in terms of, for example, diet and exercise [13,29,33]. In addition, there is evidence that supports the use of educational animations to communicate about several health-related topics [30,32,34].

A systematic review of adolescent-targeted HPV educational animations found that the animations significantly increased motivation toward receiving the HPV vaccination [30]. However, the analysis also showed that the interventions were not effective with regard to attitudes or behavior in the long term [30]. These findings indicate that more research needs to be conducted to determine how to design such messages and the effects these messages may have on individuals’ outcomes and health conversations [30,32]. In addition, more research is necessary to understand the effects these interventions can have on diverse audiences [20].

### Rationale for This Study

This study describes the development and assessment of two theory-based multimedia messages (an animated video and a game) to communicate with children about HPV and the HPV vaccine. The format and content of these messages were created based on the insights of children collected through previous formative research [35,36]. The study’s aims are as follows:

1. Understand children’s acceptability of the messages.
2. Measure the effects of the messages on children’s HPV-related outcomes.
3. Verify whether children’s confidence and willingness to communicate with their parents and health care professionals about the HPV vaccine increased after message exposure.

This study was conducted in Italy. In this country, HPV vaccination rates for middle school children are worryingly dropping. The most recent data indicate that the HPV vaccine coverage rates for females in 2015 were 66.64% and 56.26% for the first and second dose, respectively, but in 2018, the rates dropped to 61.68% and 40.36%, respectively. For males, the situation is even more severe. In 2018, the vaccination rate was very low: 44.05% for the first dose and 20.82% for the second one. Official data from 2015 are not available for males [37].

## Methods

A multidisciplinary team composed of experts in health communication, social science, and interaction design used (1) a human-centered–design approach to develop the game and the animated video and (2) a mixed methods design consisting of focus group discussions and an experiment with Italian middle school children to evaluate the effects of 2 multimedia messages (animated video and game).

### Ethics Approval

Ethics approval was received by the University of Kentucky IRB (institutional review board application number 54851).

### Participants and Recruitment

Once ethics approval was received, Italian middle school children from a large public school in north Italy were invited to join the study. Teachers distributed a leaflet and the informed consent documents to all the parents of children (aged 11-12 years) enrolled in the second year of middle school. Children whose parents signed the informed consent document were considered to have given child assent. In total, 35 middle school students participated in the project. Most of the participants were female (20/35, 57%). All the students recruited were attending the second year of middle school at the time of the study. This sampling decision was made because in Italy the HPV vaccine is provided for free to all children in their 12th year of life (aged approximately 11-12 years).

### Procedures

#### Overview

Focus groups were conducted in January 2020. All focus groups were conducted in Italian by the same moderator (AO) for purposes of consistency. Considering the number of children recruited, 9 focus groups were scheduled and randomly assigned to either the animated video or game conditions. Children recruited for this study were then randomly assigned to 1 of the 9 focus groups. After an initial ice-breaking exercise, the children were asked to complete a preintervention questionnaire. Next, they watched the animated video (20/35, 57%) or played the game (15/35, 43%). After watching the animated video or playing the game, the children were asked to complete a postintervention questionnaire. The measures used in the preintervention and postintervention questionnaires were the same. This choice allowed us to evaluate differences in responses due to being exposed to either of the 2 messages. Once all the children completed the postintervention questionnaire, the focus group discussion on the animated video or game began. The children were asked a few questions about the message they interacted with. The questions concerned (1) the purpose of the animated video and game, (2) what they thought children would like about the animated video and game, (3) what they thought children would not like about the animated video and game, (4) their opinions of the characters in the animated video and game, and (5) how (and if) they would use the animated video and game to talk with other people about health and the HPV vaccine. After this discussion, the children were invited to interact with the experimental message that they had not been randomized to (either the animated video or the game). They were then invited to discuss the same questions for the second message as well. The procedures used to analyze the focus group discussions and the experimental data are described in the following sections.

#### Questionnaire Measures

##### Knowledge

Knowledge of HPV was measured with an 8-item scale adapted from Forster et al [38]. Examples of the items included *Males cannot get HPV* (False) or *HPV can cause cancer* (True). Participants could answer on a scale ranging from 1 (strongly disagree) to 5 (strongly agree).

##### Attitudes

Attitudes were measured using a 5-point semantic differential scale with 3 pairs of opposite adjectives regarding vaccinating against HPV: bad and good, harmful and healthy, and cannot protect me from some cancers and can protect me from getting cancers. The scale showed good reliability (Cronbach *α*=.76).

##### Intention to Talk

Intention to participate in the conversations about the HPV vaccine was measured by 1 item: *I feel I will be invested in deciding whether to have the HPV vaccine.* The children could answer this item on a scale ranging from 1 (strongly disagree) to 5 (strongly agree).

##### Self-efficacy

Self-efficacy was measured using a 5-item scale from Forster et al [38]. Examples of the items included *I feel comfortable talking to my parents about whether to have the HPV vaccine* and *I feel comfortable asking my physician any questions I may have before receiving my HPV vaccination*. The scale showed medium reliability (Cronbach *α*=.50).

##### Subjective Norms

Subjective norms were measured using the 4-item scale from Forster et al [38], with each item measured on a scale ranging from 1 (strongly disagree) to 5 (strongly agree). Examples of the items included *My parents think that getting the HPV vaccine is important* and *My friends think that getting the HPV vaccine is important.* The scale showed good reliability (Cronbach *α*=.70).

##### Fear

Fear was measured using the 6-item scale from Forster et al [38]. Examples of the items included *I expect that the HPV vaccination will be very painful* and *I am worried about the side effects of the HPV vaccine.* The scale showed medium reliability (Cronbach *α*=.56).

##### Enjoyment

Enjoyment was measured with 2 items: *I had fun watching the animation* or *I had fun playing the quiz game* and *I found the animation pleasant to watch* or *I found the quiz game pleasant to play*. The children could answer each item on a scale from 1 (strongly disagree) to 5 (strongly agree). This measure was used in the postintervention questionnaire only.

##### Involvement With the Message

This construct was measured with 2 items that the children could rate on a scale from 1 to 5: *The animation I watched was important to me* or *The quiz game I played was important to me* and *The animation I watched was interesting to me* or *The quiz game I played was interesting to me*. This measure was used in the postintervention questionnaire only.

### Development of the Game and the Animated Video

#### Overview

**Figure 1:**
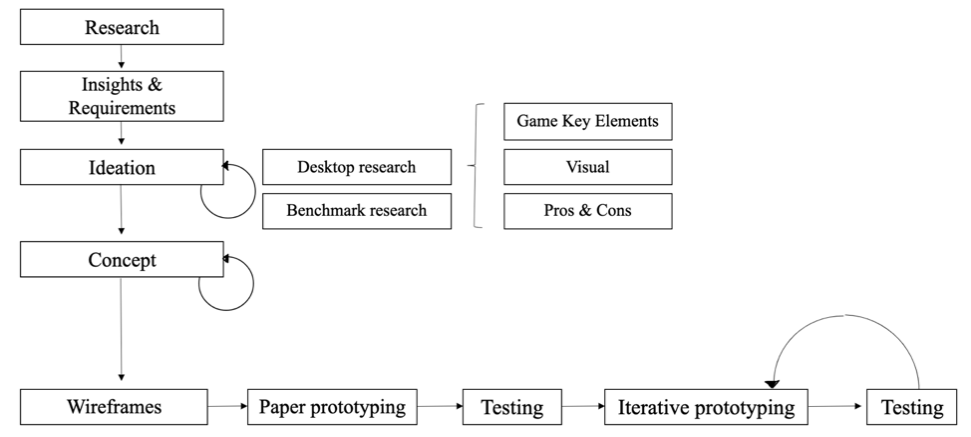
The human-centered–design approach followed in this project.

The content and format of the messages were informed by previous formative research that (1) explored Italian children’s understanding of, and concerns about, HPV and the HPV vaccine; and (2) investigated channels, sources, strategies, and characters that may be acceptable and useful to learn and talk about HPV and the HPV vaccine with their parents and health care professionals [35,36]. This formative research indicated that a promising approach to work with Italian children would be to develop multimedia materials that children could access both at school and at home. Such materials needed to focus on a few key questions the children had about HPV and the HPV vaccine to help them find an answer to key questions they identified as important [35,36]. The findings from the formative research and key constructs from the theory of planned behavior (TPB) [39], social cognitive theory (SCT) [40], and the gamification approach [41] were used to create the text and characters of the animated video and to design the logic of the game. The development of the game logic followed a human-centered–design approach, indicated in Figure 1.

The TPB [39] identifies intentions to perform a behavior as the main predictor of the behavior. To positively influence intentions, it is necessary to work on individuals’ attitudes toward the behavior, their perceived social norms, and perceived control over the behavior [39]. The SCT argues that we learn to perform a behavior by observing other people doing it and by looking at the outcomes of such behavior [40]. The gamification approach identifies several affordances that support and promote the value that an individual will assign to a game [41].

The TPB helped us to identify the key constructs to include in our game and animated video to help children engage in conversations about the HPV vaccine. We considered the insights from the SCT in designing the scenes of the animated video and game. Specifically, we showed children in the act of discussing the HPV vaccine with a health care professional, showing the positive outcomes of such a discussion and the strategies to address the challenges of engaging in such conversations.

#### Animated Video

**Figure 2:**
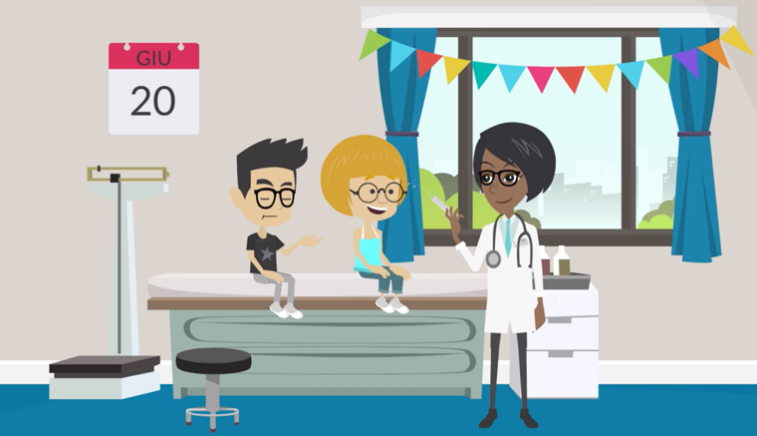
Screenshot from the animated video.

The animated video *Salute e HPV* (Health and HPV) aims to guide 2 middle school children (characters in the animated video) to discover the guidelines to be healthy. The video introduces the HPV vaccine to the children as a standard activity (such as practicing physical activity or eating a healthy diet) that they should pursue at their age to remain healthy. The video shows several scenarios in which the children practice physical activity, eat a healthy diet, and brush their teeth, along with their parents. The video also mentions the importance of sleeping at least 8 hours per night. At the end of these scenarios, the narrator (a health care professional) highlights that children also need to take the HPV vaccine to remain healthy. The choice of including this activity among several that children in Italy are typically comfortable with was made to reduce the fear and stigma associated with the HPV vaccine [37] and to stress the importance of getting vaccinated during the middle school years, as per the recommendation of the Italian ministry of health [35]. At this point in the video, the health care professional appears on screen and starts answering questions from the children about the HPV vaccine. First, the health care professional describes HPV and the diseases that it can cause. Second, the health care professional focuses on the safety of the vaccine, showing scientists in a laboratory studying and evaluating the vaccine. Third, the health care professional describes how the procedure for getting vaccinated works. The health care professional is shown talking with the 2 children, one of whom raises some concerns and explains that they are scared. The health care professional reassures them, telling them that it is common to “be scared” and to “talk about” these feelings. The health care professional also identifies some strategies the children can follow to reduce their fears. After this interaction, the children feel encouraged and receive the vaccine. Fourth, the health care professional explains that the vaccine is available for free and recommends that the children talk with their parents and physician to obtain more information. The health care professional and the children together indicate some sources that children and their parents can use to obtain more information. The video is available on YouTube [42]. Figure 2 shows a screenshot from the animated video.

#### Game

The game *Salute e HPV* (Health and HPV) was designed following the human-centered–design approach indicated in Figure 1. This approach, successfully adopted in several health-related interventions [43], helps to design interfaces and web-based platforms able to meet the needs of the audience (in this study, Italian middle school children). The approach includes several iterative phases. In the ideation phase, several insights on children’s needs and preferences were gained. We collected these insights through a series of focus groups conducted in the year before the development of the game [36,37]. After developing a greater understanding of children’s perspectives, concepts were identified by the designers in collaboration with the researchers who conducted the preliminary focus groups. The proposed solutions were then developed and improved through the refinement and testing of several prototypes [43,44]. The research team tested the prototypes multiple times before using the final prototype adopted in this study.

The game is a web-based quiz available on the web that can be played by children alone at home or in a group if at school. In this study, children played in groups of 2 because of the limited number of computers available. The game gives children the opportunity to choose a character to play with (the options are a boy or a girl, the same used in the animated video). The characters’ names were Francesco and Giulia, the most common names given to newborns the year the children in this study were born. This choice was made to help the children better identify with the characters. For the health care professional, the same character as in the animated video was used. The role of the health care professional was to provide children with correct information. Children could not choose to play with the health care professional character.

The content of the game was consistent with the content of the video. The game included the same key questions and information as in the animated video. For each of the 5 questions, the children were shown 4 pairs of statements related to the question. Each pair included 1 correct and 1 incorrect statement for the children to choose from. Once the children read the objective of the game and instructions for playing it, they could start the game. The game was designed to include several motivational affordances (Textbox 1).

**Figure 3:**
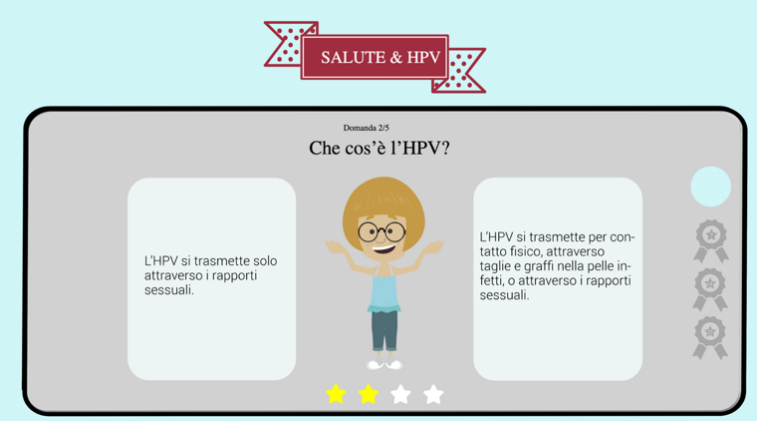
Screenshot from the web-based game. HPV: human papillomavirus.

These motivational affordances were selected because they can influence gamers in several ways. In particular, *points and achievements* and *feedback* were included in the design of the game to support children’s autonomy and competence [45]. A *clear goal* and the *progress bar* were included to provide children with a means to measure their performance [46]. Ultimately, previous research indicates that these motivational affordances can influence the enjoyment of, and engagement with, the game experienced by gamers [46]. Figure 3 shows a screenshot of the game.

**Motivational affordances included in the game.**

**Points and achievements**

• Numbers and medals were used to reward children for successfully progressing in the game

**Progress bar**

• Stars were used to show children at which point of the game they had reached

**Clear goal**

• The goal of the game was described at the beginning of the game, along with the instructions for playing the game

**Feedback**

• Children received feedback through the use of colors (green for correct answers and red for incorrect ones) and the physicians’ statement, provided at the end of each level (after each of the board’s 5 questions)

### Data Analysis

#### Focus Group Discussions

The focus group discussions were audio recorded and transcribed verbatim in their original language. The real names of the children were deleted and substituted to protect their identity. The transcripts were uploaded in NVivo (version 12), a qualitative data analysis software (QSR International). The analysis of the discussions proceeded through several steps. First, the researcher (AO) familiarized herself with the transcripts. This process included reading the transcripts several times and considering the notes taken for each of the focus groups. Second, the transcripts were coded line by line [47], following a constant comparative method [48]. Third, recurring themes were identified and further refined. Fourth, the transcripts were recoded to include the changes made for each specific category. Finally, data were compiled in brief summaries. The brief summaries are reported in the *Results* section.

#### Experiment

A series of analyses of covariance were run to verify whether the animated video and the game were effective in improving the children’s attitudes, knowledge, subjective norms, self-efficacy, intentions, and emotions regarding the HPV vaccine. Preintervention scores were inserted as covariates. Analyses of variance were run to measure the children’s enjoyment and involvement with the animated video and the game.

## Results

### Focus Group Discussions

#### Overview

Several themes emerged from the focus groups. First, the children discussed the knowledge they acquired or retained from watching the animated video or playing the game. Second, the children explained what they liked and what should be improved upon in the animated video and the game. Third, the children reflected on the characters in the animated video and the game. Finally, the children commented on how they would use the information from the animated video and the game in their lives.

#### Knowledge Acquired

After watching the animated video or playing the game, the children tried to summarize the information they remembered the most. In particular, they mentioned the association between the virus and some types of cancers, as well as the fact that the vaccine is an effective strategy to prevent cancer:

I’d heard about this vaccine, but I didn’t really know much. I knew it was transmitted through sexual intercourse and that’s all, but I didn’t know it could cause cancer.

Mia

Next, the children remembered that the vaccine is available in many countries around the world, and that in Italy, it is given for free to every child aged 12 years. According to the children, this was a very important insight that could also help parents:

This vaccination in Italy is also free of charge, so parents may be less worried about possible costs.

Luke

#### Features Children Liked

The children indicated several features of the video and the game that they liked. For both the animated video and the game, they mentioned the colors, which they described as “bright” and “attractive”:

The designs are very clear but are not exaggerated, they are stylized, beautifully colored.

Marzia

The children also commented on the strategies used to present the information. In particular, for the animated video, the children appreciated the use of the *whiteboard* to illustrate the concepts presented by the health care professional. They specifically liked the fact that both the health care professional and the images of what she was describing were shown at the same time, and this aided their comprehension:

It’s nice that there’s the tarp [whiteboard] with the projector.

Lia

It’s very detailed.

Peter

You see the images at the same time as the doctor explaining, there’s not just the voice.

Lia

Regarding the game, they appreciated the summary of the information provided at the end of each question. They felt that they could still learn from the game even if they did not know much about HPV or the HPV vaccine before playing it:

It’s pretty clearly explained. It’s nice that every time it asks you a question, you get the summary afterward. Even if you haven’t seen the video, maybe you can get most of the answers wrong, but then with the summary, you understand.

Thomas

In the end, when asked which of the 2 materials they preferred, the children said that they liked the animated video and the game equally. They said that children should use both these educational materials because, although they included the same information, they felt that the 2 materials complemented each other:

They are two things that talk about the same subject, but they are different because in the game you had to think about what you understood, whereas the video serves to confirm your hypothesis.

Azzurra

#### Features That Need Improvement

The children recommended that a few elements could be improved in both the game and the animated video. In particular, the children felt that there was some missing information:

The length [of the video] is right, but maybe you could add a part explaining what the consequences are if you don’t get this vaccine.

Tia

More specifically, the children indicated that they wished they had been able to learn more about the possible side effects of the vaccine as well as the symptoms of an HPV infection, besides the risk of getting cancer:

Always add, as in the video, always add what can happen if you don’t get the vaccine.

Marzia

And what are the side effects.

Tia

Also because we could convince more people that this infection isn’t a small thing, so it’s much better to prevent it with the vaccine.

Marzia

#### Characters

Overall, the children liked the characters in both the animated video and the game for several reasons. The most liked character was the health care professional, appreciated for both her caring attitude and for the type of information she provided:

I [liked] the doctor because even [though] the children are afraid of the needle, she advises them to close their eyes, she’s thoughtful.

Lia

She also gives them advice on how to feel less pain, have less anxiety.

Peter

The children also liked the 2 young characters, both their design as well as their personality. Regarding the overall design, some children also mentioned the presence of diversity as a positive characteristic that they valued and helped them to better identify with the characters:

I liked that the children were of different ethnicities.

Tobias

She [the doctor] looks like me!

Mara

Regarding the personalities of the characters, one of the moments the children appreciated the characters the most in the animated video was when the children visit the physician’s office to receive the vaccine, and 2 different emotional reactions were displayed:

Then the characters are nice, they make the idea. The boy was worried about the vaccination while the girl wasn’t, it indicates that there are different kinds of people.

Luke

A negative comment about the characters concerned the size of the children. For some of the children, this contributed to making the characters look as though they were younger than the children:

Maybe looking at them like that, the characters being too short may look like children. They don’t look like children my age to me.

Philip

#### Use of the Animated Video and Game

The children mentioned that one of the most interesting elements they liked about the video and the game was that they could use these tools to learn more about HPV and the HPV vaccine:

This game is instructive. It can be the litmus test [literally prova del nove (test of 9) in Italian] to see if you understand.

Duccio

It’s like some kind of test, and it makes you realize what you’ve figured out but it doesn’t make you feel anxious at least.

Agata

The children expressed the desire to talk more about these topics with adults as well as with their friends. This concept emerged consistently in each of the focus groups, either explicitly or in the form of the questions asked by the children. For example, the children asked whether they could show the video to their parents or have a say in the decision to receive the HPV vaccine:

But is it something that parents choose, or can I choose too [to get the vaccine]?

Elia

What do you think?

Moderator

I’d like to make the decision too, because it’s about me, and if I want to do it, I can do it.

Elia

The children consistently mentioned that the video could be a means to facilitate the discussion with the parents. It could be something that parents could use to talk to their children about HPV:

It could be good for the parents, too. You say there’s this disease, it can also cause tumors, my daughter has to be cured, I have to give her vaccinations. So, it might help the conversation a little bit.

Azzurra

The children also wished that the video and the game were available on the web on several social media platforms so that both they and their parents could access them easily:

Can we find it online? To show it to our parents?

Sarah

I would go to see the video on YouTube, as a platform that would be great, but also Instagram and Facebook, where there are many adults who could make the decision to have their children vaccinated.

Maria

### Experiment

Several analyses of covariance were run to understand the effects of the messages assessed in this study. The dependent variables used were, in turn, postintervention knowledge, attitude, intention, self-efficacy, subjective norms, and fear scores. The independent variable was the modality used (animated video vs game). Participants’ preintervention scores were used as covariates. Both the animated video and the game were effective in changing some of the key variables explored to improve children’s knowledge and perceptions of the HPV vaccine, but any single message was not statistically more effective than the others. For the animated video, significant changes from pre- to postintervention scores were observed for most of the variables assessed, with the exclusion of attitude and subjective norms. For the game, the only statistically significant change from pre- to postmessage exposure was observed for knowledge and intention. The results for each of the variables for both the animated video and the game are shown in Table 1.

These results reinforce what emerged from the focus groups data: the children retained the information from both educational strategies (animated video and game) and enjoyed watching, and interacting with, them.

**Table 1:** Results and descriptive statistics for study variables by experimental condition (N=35).

Condition			Time point														*t* test (*df*)					*P* value				Mean difference	
			Before the intervention						After the intervention																		
			Mean (SD)		n (%)			Mean (SD)				n (%)															
**Animated video, n=20**																											
	Knowledge	3.34 (0.36)				18 (90)				3.90 (0.37)			18 (90)					6.06 (17)	<.001^a^								0.55
	Attitude	3.97 (0.94)				20 (100)				4.12 (1.23)			20 (100)					0.81 (19)	.42								0.15
	Intention	3.20 (1.19)				20 (100)				3.80 (0.83)			20 (100)					3.94 (19)	<.001^a^								0.60
	Self-efficacy	3.63 (0.51)				19 (95)				3.88 (0.37)			19 (95)					3.13 (18)	<.001^a^								0.25
	Subjective norms	3.86 (0.63)				20 (100)				4.01 (0.52)			20 (100)					1.35 (19)	.19								0.15
	Fears	2.94 (0.49)				20 (100)				2.69 (0.50)			20 (100)					–3.96 (19)	<.001^a^								–0.25
	Enjoyment	N/A^b^				N/A				4.45 (0.39)			20 (100)					N/A (19)	.29^c^								N/A
	Message involvement	N/A				N/A				4.37 (0.48)			20 (100)					N/A (19)	.72^c^								N/A
**Game, n=15**																											
	Knowledge	3.34 (0.51)		13 (87)			3.79 (0.35)				13 (87)			3.13 (12)					<.001^a^				0.45				
	Attitude	4.38 (0.66)		15 (100)			4.47 (0.53)				15 (100)			1.00 (14)					.33				0.08				
	Intention	2.93 (1.10)		15 (100)			3.73 (0.70)				15 (100)			2.86 (14)					.01^a^				0.80				
	Self-efficacy	3.72 (0.54)		15 (100)			3.75 (0.56)				15 (100)			0.24 (14)					.81				0.03				
	Subjective norms	3.40 (0.51)		15 (100)			3.62 (0.67)				15 (100)			1.94 (14)					.07				0.22				
	Fears	2.76 (0.55)		15 (100)			2.71 (0.53)				15 (100)			–0.69 (14)					.49				–0.05				
	Enjoyment	N/A		N/A			4.60 (0.43)				15 (100)			N/A (14)					.29^c^				N/A				
	Message involvement	N/A		N/A			4.43 (0.45)				15 (100)			N/A (14)					.72^c^				N/A				

^a^*P*<.05.

^b^N/A: not applicable.

^c^Information pertaining to the comparison of animated video and game.

## Discussion

### Principal Findings

This study adopted a mixed methods approach consisting of focus group discussions and an embedded experiment to assess the feasibility of 2 educational multimedia materials on HPV and the HPV vaccination. The purpose of these educational materials was to educate children and provide them with the skills to discuss these topics with their parents and health care professionals. Both the qualitative findings and the results from the experiment indicated that the 2 educational materials were well received and improved children’s intention to discuss the HPV vaccine from pre- to postmessage exposure. Children liked the characters presented in the animated video and the game and provided several suggestions on how to improve these materials. By educating children on the HPV vaccine, researchers and practitioners have the potential to aid in the promotion of personal health decision-making, strengthening communication about personal health, and enhancing personal health behaviors, including developing more favorable attitudes toward receiving the HPV vaccine [7].

This study took place in Italy, a country where the field of health communication is still underdeveloped, and it represents a pioneering effort to engage and represent a relatively understudied audience: Italian children. The study provides evidence in support of the use of multimedia interventions such as animated videos and web-based games to improve child-parent communication and HPV-related discussions. It indicates how communication and behavior change theories can be integrated into the human-centered–design approach. It also describes the working process used by the interdisciplinary team of communication scholars and interaction designers who created and evaluated the educational multimedia materials.

This study suggests that animated videos and web-based games can be effective multimedia strategies to improve children’s knowledge about the HPV vaccination and their intention to discuss their health, in particular the HPV vaccine, with their parents and health care professionals. During the focus group discussions, the children indicated the features they appreciated in both the animated video and the game, which included in particular the personality and caring attitude of the characters. This finding suggests that showing positive examples of communication exchanges may be important to help children feel comfortable when discussing the HPV vaccine and looking for health-related information. The children also expressed the desire to show the 2 messages to their parents and friends, indicating that multimedia messages should be easily retrievable and shareable in possible future campaigns to support children’s discussion and information needs.

The experiment allowed observation not only of the improvements in knowledge of HPV and the HPV vaccine but also of the different changes in the outcome variables, depending on the message the children interacted with. When considering the emotional reaction to the vaccine, the animated video seemed to have an advantage over the web-based game. It is possible that, by showing enacted behaviors, animations may reduce children’s fear while improving their self-efficacy with regard to health discussions. These findings are significant, considering that children encounter several challenges to engaging in conversations about their health, particularly when such discussions include a sexual-education component [49,50]. It is important to note that the children participating in this study felt that the video and game complemented each other, and that by using both, they were able to learn and test their knowledge. The children also suggested that the animated video and the web-based game would also be helpful to parents in providing an explanation of HPV to their children.

### Limitations and Future Directions

There are some limitations to this study that should be mentioned. First, data were collected at only 1 school in the northern part of Italy. It is possible that children from different areas of Italy would have different perceptions and previous experiences that may affect how they receive the animated video and game. Second, parents’ and physicians’ comments about the game and animated video were not collected, limiting our ability to contextualize the children’s experiences. Third, the scales used in this study were translated from English to Italian, but the translated questionnaire had not been previously validated with an Italian audience.

Future research is necessary to further extend this preliminary investigation to control for the effects of the specific features of the game by increasing the number of children exposed to these educational multimedia materials. A greater sample of children would allow researchers to evaluate the possible role of mediators and moderators to explain the effects of the animated video and the game to improve children’s self-efficacy and attitudes. Future studies should also aim to measure improvements in the conversations between parents and their children resulting from the exposure to the game and the animated video through observations of dyadic conversations. Furthermore, it would be beneficial to understand whether the animated video and game are useful tools to help parents begin a discussion with their child about HPV. Future studies should also better explore the effects that educational multimedia messages have on children’s conversations with their teachers and peers to inform the design of school-based interventions. Future studies should also control for the HPV vaccination status of the children and consider targeting the messages to it. Ultimately, future studies should integrate and evaluate these materials and educational modules in comprehensive health education packages that include information on several health issues relevant to children aged 11-12 years.

### Conclusions

Engaging children in discussions about their health in general and HPV and the HPV vaccine in particular is important for empowering children and meeting their information needs. The animated video and web-based game evaluated in this study were well received by the children and were shown to be promising messages to improve several outcomes. We hope that the findings of, and procedures used in, this study will inspire other researchers to continue this line of research and further identify and develop strategies and campaigns to engage children in their health care.

## Abbreviations

HPV: human papillomavirus
SCT: social cognitive theory
TPB: theory of planned behavior
